# A new algorithm to train hidden Markov models for biological sequences with partial labels

**DOI:** 10.1186/s12859-021-04080-0

**Published:** 2021-03-26

**Authors:** Jiefu Li, Jung-Youn Lee, Li Liao

**Affiliations:** 1 Computer and Information Sciences, University of Delaware, 101 Smith Hall, Newark, DE 19716 USA; 2 Plant and Soil Sciences, University of Delaware, 15 Innovation Way, Newark, 19716 USA; 3 Delaware Biotechnology Institute, University of Delaware, 15 Innovation Way, Newark, 19716 USA; 4 Data Science Institute, University of Delaware, 100 Discovery Blvd, Newark, 19713 USA

**Keywords:** Hidden Markov model, Partial label, Constrained Baum-Welch algorithm, Biological sequences

## Abstract

**Background:**

Hidden Markov models (HMM) are a powerful tool for analyzing biological sequences in a wide variety of applications, from profiling functional protein families to identifying functional domains. The standard method used for HMM training is either by maximum likelihood using counting when sequences are labelled or by expectation maximization, such as the Baum–Welch algorithm, when sequences are unlabelled. However, increasingly there are situations where sequences are just partially labelled. In this paper, we designed a new training method based on the Baum–Welch algorithm to train HMMs for situations in which only partial labeling is available for certain biological problems.

**Results:**

Compared with a similar method previously reported that is designed for the purpose of active learning in text mining, our method achieves significant improvements in model training, as demonstrated by higher accuracy when the trained models are tested for decoding with both synthetic data and real data.

**Conclusions:**

A novel training method is developed to improve the training of hidden Markov models by utilizing partial labelled data. The method will impact on detecting de novo motifs and signals in biological sequence data. In particular, the method will be deployed in active learning mode to the ongoing research in detecting plasmodesmata targeting signals and assess the performance with validations from wet-lab experiments.

## Background

Hidden Markov model [1–5] is a well known probabilistic model in the field of machine learning, suitable for detecting patterns in sequential data, such as plain texts, biological sequences, and time series data in the stock market. For all these applications, successful learning depends, to a large degree, on the amount and, more importantly, the quality of the data. In text mining problem, though the data amount is huge, careful labelling tasks consume massive human labor [6]. In biological sequence analysis, discovering de novo signal remains challenging because a precise full labeling via wet-lab experiments demand even more resources and time, and hence it is considered unfeasible in general. Therefore, research is necessary in data handling with different labelling quality for applied machine learning community. In this paper, we focus on designing a Baum–Welch-algorithm based learning method for HMMs to handle the biological problems when only partial labeling is available in training data.

This work is inspired by our recent research on detecting de novo plasmodesmata targeting signals in Arabidopsis Plasmodesmata-located proteins (PDLPs). PDLPs are type I transmembrane proteins, which are targeted to intercellular pores called plasmodesmata that form at the cellular junctions in plants [7]. In our study [8], by building a 3-state HMM, we predicted the presence of two different plasmodesmata targeting signals (named alpha and beta) in the juxta membrane region of PDLPs. While all the predicted signals were successfully verified in wet-lab experiments so far, some predicted signals contain residues that do not conform to the true signal; wet-lab experiments showed that those residues alone was not sufficient to target the protein to plasmodesmata. Because both the cost and time are high for wet-lab experiments, an improved HMM would be highly desirable. However, due to the limitation in the number of the training examples–Arabidopsis genome encodes only eight PDLP members, further improvements of the model can be hardly achieved. It would require to fully utilize the current wet-lab experimental results to train the model, i.e., by labeling the residues that have been already shown to be either part of the signals or not part of the signals, given that labels are not available for all the residues due to limited experimental results.

In a related work by Tamposis et al., a semi-supervised approach is developed to handle a mixture of training sequences that contains a subset of fully labelled sequences, with the remaining sequences having no labels at all or partial labels [9]. Their method uses the fully labelled sequences to train the parameters for HMMs and then use Viterbi algorithm to predict the missing labels followed by training the model again with the predicted labels. This process is iterated until a convergence condition is met. Instead, we are specifically interested in situations where no fully labelled sequences are available and often the partial labeling is also sparse. In the text mining field, HMM training algorithm of handling partial label was developed especially for active learning purposes and designed to fit into text mining special situation: no label scenario, or in other words, no meaningful label can be assigned [6]. However the unit of observation in text mining and information retrieval is a word, instead of a single letter, corresponding to individual amino acid residue as in biological sequences. So, in order to deal with the partial labeling aforementioned, we have designed a novel Baum–Welch based HMM training algorithm to leverage partial label information with techniques of model selection through partial labels. Besides the difference in the observation unit, our algorithm differs from [6] primarily in how to calculate the expected value for a given partial label at a given position: our method sums over hidden state paths that must be subject to constraints anywhere given partial labels in the training sequence. In contrast, in [6] the expected value for a given partial label at a given position is calculated by summing over paths that are only constrained at the position being considered, and anywhere else in the sequence the hidden paths are free to go through all possible states (labels) even at positions where partial labels are given. Moreover, this difference affects how the expected value for a transition is calculated, regardless whether the transition happens to involve one partial label, two partial labels, or no partial labels at all. The comparison between our method and the method described in [6] showed that our method outperformed in both synthetic and real data for decoding task in biological problems.

The rest of this paper is organized as follows. First, the relevant background knowledge of HMM is briefly reviewed, and notations are introduced. Then, our method of training HMM when only partial label sequences are available is described in details. This is followed with experiments and results to examine and demonstrate the modelling power of the novel algorithm. Discussion and conclusion are given at the end.

## Methods

### Hidden Markov model review

In general, a HMM consists of a set of states $S_i$, $i = 1 \,{\text{to}}\, N$, and a set of alphabets *K* that can be emitted from these states with various frequencies; $b_j(k)$ stands for the frequency of letter $k \in K$ being emitted from state $S_j$, and we use *B* to denote the emission matrix of dimension $N \times K$, containing $b_j(k)$ as elements. Transitions among states can be depicted as a graph, often referred as model architecture or model structure: each state is represented as a node, and transition from state $S_i$ to state $S_j$ is represented by a directed edge, with a weight $a_{ij}$ being the transition probability, and we use *A* to denote the transition matrix of dimension $N \times N$, containing $a_{ij}$ as elements. Hereafter, we often refer to a state $S_i$ by its index *i*.

Given a HMM, let $\theta$ stand for collectively all its parameters, namely the emission frequencies $b_j(k)$ and transition probabilities $a_{ij}$. Given a sequence of observation *O*, and its elements $O_t \in K$, where $t = 1\ldots T$, a main assumption of using HMM is that each letter in the sequence is emitted from a state of the model, so correspondingly there is a state sequence, forming a Markov chain, which is hidden from direct observation, hence the name: hidden Markov model. One task (decoding) is, therefore, to find the most probable state sequence (also called hidden path) $X^*$ : $X^* = {{\,\mathrm{argmax}\,}}_{X} Pr(O,X |\theta )$, among all possible state sequences that can emit the observation sequence *O*. The second task is to train the model on a set of *m* training sequences. This task is accomplished by adjusting model parameters $\theta$ to maximize the likelihood $\sum _{s=1}^{m} Pr(O^s |\theta )$ of observing the given training sequences $O^{s}$, where $s = 1\ldots m$ [10].

The decoding task is well studied and straightforward and is solved by Viterbi algorithm efficiently [11]. The technique guarantees to return the optimal answer. Note that, in the work by Bagos et al. [12], a modified Viterbi algorithm is developed to incorporate prior topological information as partial labels to improve predictions, whereas our focus is instead on how to use the partial labels in training the model. However, the second task, or the training of a HMM is not guaranteed to reach optimum when labels are not given for the training sequences.

The major training algorithms of HMM are the following three in general: maximum likelihood, Baum–Welch algorithm, and Viterbi training [13]. Maximum likelihood is used when label information is available fully, and it returns the optimal solution. The latter two algorithms are used when no label information is available. Interested readers can find a gentle introduction and tutorial for hidden Markov models in [10]. For the purposes of comparison, we adopt notations in [6] for future discussion of both the background knowledge and our method. The description of notations is shown in Table 1.

**Table 1:** Notations

Symbols	Explanations
$\theta$	Hidden Markov model: $\theta = (\pi , A,B)$
*N*	States’ number in hidden Markov model
*K*	Symbolic Number in hidden Markov model
*A*	Transition matrix with dimension $N \times N$
$a_{ij}$	Probability of state i transition to state j
*B*	Emission matrix with dimension $N \times K$
$b_{j}(k)$	Probability of state j emitted from symbol k
$\pi$	Initial probability of states with dimension $N \times 1$
$O^s$	The *s*th sequence with length $T^s$
$X^{s}$	State sequence of $O^s$
*m*	Total number of sequences

In this paper, we focus on a special case for training HMMs when only partial label is available. Or in other words, we aimed at finding model $\theta$ so that $Pr(O |\theta )$ is maximized (locally) and the resulting decoded state sequence must satisfy the partial labels given in the training sequences at the same time.

### Training hidden Markov model with partial label sequences

As introduced in the previous section, when no labels are available, Baum–Welch algorithm is typically used to train HMM and Viterbi training is sometimes used for speed and simplicity; when all label information is given, training HMM is straight forward by maximum likelihood approach. Currently, training HMM with partial label is mainly studied in the field of text mining, with a particular focus on active learning problems, such as the work done in [6], with which we compare our proposed method.

Our proposed method is a novel approach to this partial label training problem with modification of Baum–Welch algorithm (called constrained Baum–Welch algorithm) and a model selection technique, which helps our algorithm leverage available information and improve the training and performance in decoding task. In the next two subsections, we discuss in detail our constrained Baum–Welch algorithm and the model selection methods respectively and how to combine the two for model training.

#### Constrained Baum–Welch algorithm

The standard Baum–Welch algorithm is an Expectation-Maximization approach to maximizing likelihood when the system contains latent variables, which are the state sequences for hidden Markov models when training sequences are not labelled. Our constrained Baum–Welch algorithm (cBW) is similar to the standard Baum–Welch algorithm except that the training sequences are partially labelled, which imposes the constraints on the possible hidden state paths in calculating the expectation. Standard Baum–Welch algorithm is divided into E-step and M-step. The M-step of cBW algorithm is identical to standard Baum–Welch’s. The difference is the E-step, computing forward and backward matrices. The forward matrix $\alpha$ is of $N \times T$, where *N* is the number of states and *T* is the sequence length. An element $\alpha _i(t)$ is the probability of the observed sequence up to and including $O_t$, with the symbol $O_t$ being emitted from state *i*. The backward matrix $\beta$ is of $N \times T$ dimension has element $\beta _i(t)$ as the probability of the observed sequence from position *t* onto the end, with the symbol $O_t$ being emitted from state *i*. The formulas of computing $\alpha$ and $\beta$ are shown as following respectively.

Given the model $\theta = (\pi , A,B)$, where $\pi$ is a *N* dimension vector, with $\pi _i$ being the probability that any hidden state path would start with state *i*. Then, the initial values of forward matrix $\alpha$ for one given training sequence $O = (O_1, \ldots , O_T)$ is computed as follows.

1 $$\begin{aligned} \alpha _i(1) = \pi b_i(O_1) \end{aligned}$$

After calculating the initial values of $\alpha$, by dynamic programming, the remaining values at any position for any state are calculated recursively by summing over the possible state paths $X = (X_1,\ldots ,X_T)$, allowed by the model, that lead to the point whose $\alpha$ value is being calculated. However, since we now have partial labels for the training sequence *O*, care must be taken to satisfy the constraints at each position $O_t$ imposed by the partial label, $L(O_t) \in S \cup \{0\}$, where a value zero means no label available. Specifically,

2 $$\begin{aligned} \alpha _i(t+1) = {\left\{ \begin{array}{ll} b_i(O_{t+1})\sum _{j =1 }^{N} \alpha _j(t) a_{ji} , &{} \quad {\text {if}}\, L(O_{t+1}) = 0 \quad {\text {or}}\quad i \\ 0 &{} \quad {\text {if}}\, L(O_{t+1}) \ne 0 \quad {\text {and}}\quad L(O_{t+1}) \ne i \\ \end{array}\right. } \end{aligned}$$

In the above equation, the first case is when position $O_{t+1}$ is either unconstrained (0) or constrained to be state *i* by the partial label. In such a case, the $\alpha$ value is computed in the same way as the standard Baum–Welch algorithm, though the actual value can still be affected by partial labels at earlier positions via recursion. The second case is when the position $t+1$ is constrained by the partial label to be a state other than *i*. In this case, $\alpha _i(t+1) = 0$. This latter case is what makes the algorithm different from the standard Baum–Welch algorithm in order to “honor” the partial labels. The backward matrix $\beta$ is initialized as the following.

3 $$\begin{aligned} \beta _i(T) = 1 \end{aligned}$$

Then, similarly, a recursive procedure is applied for the remaining of backward matrix.

4 $$\begin{aligned} \beta _i(t) = {\left\{ \begin{array}{ll} \sum _{j=1}^{N} \beta _j(t+1) a_{ij} b_j(O(t+1)), &{} \quad {\text {if}}\, L(O_{t}) = 0 \quad {\text {or}}\quad i \\ 0 &{} \quad {\text {if}}\, L(O_{t}) \ne 0 \quad {\text {and}}\quad L(O_t) \ne i \\ \end{array}\right. } \end{aligned}$$

Note that, while the $\alpha$ is calculated the same way as the modified Forward algorithm in [12] but the $\beta$ is calculated differently from their modified Backward algorithm. After the calculations of $\alpha$ and $\beta$, then we can calculate $\gamma$ variable, where $\gamma _i(t)$ is the probability of observing the training sequence *O* from all possible state paths that are allowed by hidden Markov model $\theta$ as constrained by the partial labels and go through state i at position *t*. $\gamma _i(t)$ is computed as follows.

5 $$\begin{aligned} \gamma _i(t)&= P(X(t) = i |\theta ,O) = \frac{ P(X(t) = i, O |\theta ) }{ P(O |\theta ) } \nonumber \\&= \frac{ \alpha _i(t) \beta _i(t)}{ \sum _{j = 1}^{N} \alpha _j(t) \beta _j(t) } \end{aligned}$$

where the last equal sign holds because $P(O |\theta ) = \sum _{j = 1}^{N} \alpha _j(t) \beta _j(t)$. The next step is to compute $\xi _{ij}(t)$, which is the probability of of observing the training sequence *O* from all possible state paths that are allowed by hidden Markov model $\theta$ as constrained by the partial labels and go through state *i* at positive *t* and transition to state *j* at position $t+1$:

6 $$\begin{aligned} \xi _{ij}(t)&= \frac{ P(X(t) = i,X(t+1) = j, O |\theta ) }{ P(O |\theta ) } \nonumber \\&= \frac{ \alpha _i(t) a_{ij} \beta _{j}(t+1) b_j(O(t+1))}{ P(O |\theta ) } \end{aligned}$$

Finally, with $\gamma$, $\xi$, the M-step is to update the initial probability $\pi ^*$, every elements of the transition matrix $A^*$: $a_{ij}^*$, and every elements of the emission matrix $B^*$: $b_{i}^{*}(o_k)$.

7 $$\begin{aligned} \pi (i)^*&= \gamma _i(1) \end{aligned}$$

8 $$\begin{aligned} a_{ij}^*&= \frac{ \sum _{t = 1}^{T-1} \xi _{ij}(t) }{ \sum _{t = 1}^{T-1} \gamma _i(t) } \end{aligned}$$

9 $$\begin{aligned} b_{i}^{*}(o_k)&= \frac{ \sum _{t = 1}^{T-1} \gamma _i(t) I_{O(t) = o_k} }{ \sum _{t = 1}^{T-1} \gamma _i(t) } \end{aligned}$$

where $I_{O(t) = o_k}$ stands for indicator function, which equals to 1 if $O(t) = o_k$, and 0 otherwise. Then, for the case of multiple sequences, each sequences indexed by *s*, total number of sequences of *m*, The only changing is the updating of $\pi ^*$,$A^*$, and $B^*$ as follows.

10 $$\begin{aligned} \pi (i)^*&= \frac{ \sum _{s = 1}^{m} \gamma _{i}^{s}(1)}{ m } \end{aligned}$$

11 $$\begin{aligned} a_{ij}^*&= \frac{ \sum _{s = 1}^{m} \sum _{t = 1}^{T^s-1} \xi _{ij}^{s}(t) }{ \sum _{s = 1}^{m} \sum _{t = 1}^{T^s-1} \gamma _i^{s}(t) }\end{aligned}$$

12 $$\begin{aligned} b_{i}^{*}(o_k)&= \frac{ \sum _{s = 1}^{m} \sum _{t = 1}^{T^s-1} \gamma _{i}^{s}(t) I_{O^{s}(t) = o_k} }{ \sum _{s = 1}^{m} \sum _{t = 1}^{T^s-1} \gamma _i^{s}(t) } \end{aligned}$$

**Fig. 1:**
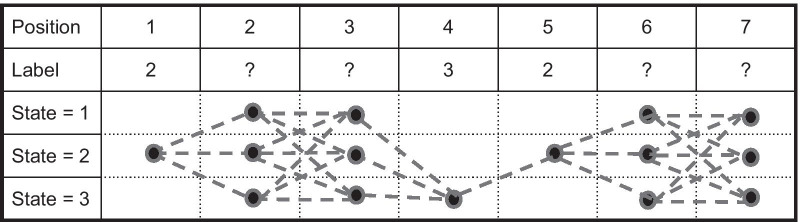
Example of constrained Baum–Welch’s forward/backward dynamic programming table construction Position 1–2 is the case of labelled position to unlabelled position. Position 3–4 is the case of unlabelled position to labelled position. Position 4–5 is the case of labelled position to labelled position. Dashed lines indicate state transitions. Generated by Google Drawings

The procedure above is repeated till either the $\sum _{s}^{m} log(P(O^s|\theta ))$ converge or reaching maximum iteration numbers set by the user. As mentioned in the Introduction section, a key difference between our method and [6] lies in the E-step for calculating the expected value for a given emission or transition. Our method handles the partial label constraints recursively for the $\alpha$ and $\beta$, whereas [6] calculates $\alpha$ and $\beta$ without using the partial labels and only uses the partial labels in resetting $\gamma$ at each partial labelled position independently, as if partial labels elsewhere would have no effect for the position being considered. Since E-step in Baum–Welch algorithm invokes forward and backward algorithms, which are essentially a dynamic programming to more efficiently calculate the likelihood: $Pr(O |\theta )$ = $\Sigma _{X\in \Gamma } Pr(O,X |\theta )$ with $\Gamma$ being the set of all hidden paths, and hence should give the same result when the likelihood is computed by exhaustively summing over probability for all possible hidden state paths. Therefore, we believe, the partial labels would restrict the possible hidden state paths, $Pr(O |\theta )$ = $\Sigma _{X \in \Gamma '} Pr(O,X |\theta )$ with $\Gamma '$ being the set of all hidden paths constrained by partial labels and such constraints should be handled recursively in the dynamic programming. Figure 1 shows an example for the forward/backward dynamic programming table construction. Another advantage of our method comparing with the method in [6] is that our training method can keep the topology of the initial transition and emission guesses for the model as standard Baum–Welch does. In other words, if prior knowledge is available for the model topology, our training method for partial label data can keep the knowledge to the end of training.

#### Model selection based on partial label information

The second part of our method is model selection based on partial label information. The rationale is straightforward: while the constrained Baum–Welch algorithm increases the log-likelihood of the given training sequences (with partial labels), iteration after iteration monotonically as ensured by EM approach, there is no direct guarantee that the increased log-likelihood would necessarily lead to higher decoding accuracy. Therefore, at each iteration of constrained Baum–Welch algorithm, decoding accuracy for the partially labelled training sequence can be calculated and factored into model selection.

Specifically, after reaching convergence condition or maximum number of iterations, the total number of iteration is *Q* and the $i^{th}$ iteration’s model and the corresponding log-likelihood can be denoted as $\theta _i$ and $\sum _{s}^{m} Log(P(O^{s}|\theta _i))$ respectively and let the decoding accuracy denote as $Accuracy(\theta _i,O,X)$. The final model returned by the algorithm can be expressed as:

13 $$\begin{aligned} {{\,\mathrm{argmin}\,}}_{\theta ^{*}} Pr(O | \theta ^{*} \equiv \{{{\,\mathrm{argmax}\,}}_{\theta _i \in \theta _{1\ldots Q}} Accuracy(\theta _i,O,X)\}) \end{aligned}$$

Notice that $\theta ^{*}$ is a set of models in general. Finally, combining the constrained Baum–Welch and the model selection described above, the overall algorithm of our proposed method is given in Algorithm 1. In next section, Tables 2, 3, 4 and 5 will show the usefulness of both this model selection method and the ability of keeping correct topology of cBW method. 
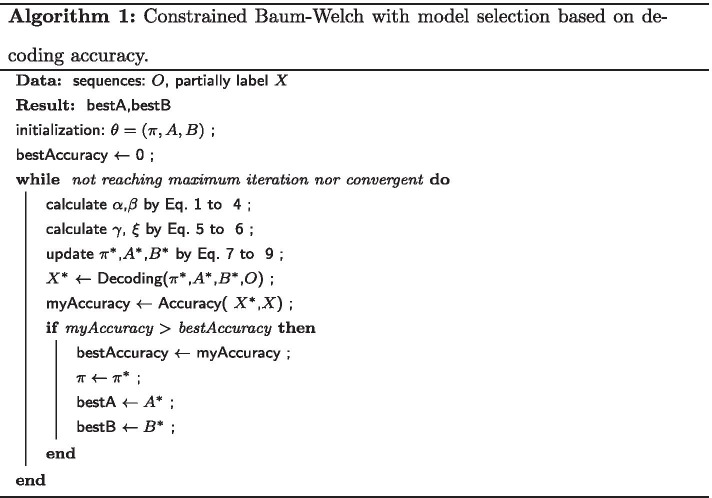

**Table 2:** Improvements of cBW + model selection, cBW alon vs Scheffer et al., with fully connected initial transition matrix for synthetic data with Viterbi algorithm

State #/training sample	Average improvements of cBW + model selection in setting 1/2	Average *p* value of cBW + model selection in setting 1/2	Average improvements of cBW alone in setting 1/2
3/1600	7.35/8.29%	2.1E−02/6.2E−05	7.61/8.49%
3/2000	7.99/8.82%	3.9E−02/2.6E−09	8.31/9.00%
3/2400	8.47/9.13%	2.8E−03/2.4E−10	8.71/9.18%
3/2800	8.51/9.24%	5.8E−03/6.9E−10	8.65/9.24%
5/1600	12.97/14.75%	7.8E−05/3.3E−05	11.13/12.82%
5/2000	14.63/16.32%	2.5E−03/1.2E−06	12.65/14.11%
5/2400	14.69/16.54%	7.8E−04/6.2E−06	12.73/14.50%
5/2800	15.56/17.22%	1.6E−02/1.3E−07	13.72/15.22%
7/1600	8.61/10.42%	3.5E−02/1.1E−02	5.56/7.20%
7/2000	10.56/12.40%	6.4E−03/6.2E−03	7.87/9.52%
7/2400	11.35/13.21%	8.2E−03/7.8E−03	8.71/10.43%
7/2800	12.16/14.06%	1.0E−03/1.1E−04	9.68/11.41%

**Table 3:** Improvements of cBW + model selection, cBW alone vs Scheffer et al. in cases of correct initial transition matrix for synthetic data with Viterbi algorithm

State #/training sample	Average improvements of cBW + model selection in setting 1/2	Average *p* value of cBW + model selection in setting 1/2	Average improvements of cBW alone in setting 1/2
3/1600	8.07/8.57%	2.3E−04/4.7E−07	8.11/8.56%
3/2000	8.58/9.05%	1.9E−06/6.5E−09	8.63/9.03%
3/2400	8.93/9.24%	1.6E−07/1.0E−09	8.97/9.20%
3/2800	8.87/9.31%	1.3E−08/1.5E−09	8.94/9.26%
5/1600	11.99/13.24%	1.7E−02/4.5E−06	11.76/13.08%
5/2000	13.07/14.20%	4.1E−02/3.4E−06	12.87/14.11%
5/2400	13.22/14.59%	2.0E−02/1.2E−05	12.94/14.35%
5/2800	13.89/15.20%	4.1E−02/1.6E−07	13.85/15.16%
7/1600	7.85/9.37%	6.7E−02/3.5E−02	6.04/7.34%
7/2000	9.75/11.28%	5.6E−03/4.1E−03	7.93/9.32%
7/2400	10.50/12.10%	1.8E−02/1.9E−02	8.99/10.53%
7/2800	11.39/12.95%	1.4E−03/1.9E−04	9.75/11.29%

**Table 4:** Improvements of cBW + model selection, cBW alon vs Scheffer et al., with fully connected initial transition matrix for synthetic data with posterior-Viterbi algorithm

State #/training sample	Average improvements of cBW + model selection in setting 1/2	Average *p* value of cBW + model selection in setting 1/2	Average improvements of cBW alone in setting 1/2
3/1600	7.08/8.02%	3.6E−02/1.9E−04	7.49/8.35%
3/2000	7.93/8.57%	9.5E−03/3.9E−05	8.32/8.88%
3/2400	8.21/8.85%	1.7E−03/2.5E−05	8.55/9.03%
3/2800	8.43/9.15%	1.8E−03/1.1E−06	8.82/9.36%
5/1600	9.13/10.62%	2.4E−02/3.2E−03	9.08/10.58%
5/2000	10.32/11.68%	1.4E−02/6.2E−05	10.38/11.76%
5/2400	11.26/12.74%	1.1E−02/2.0E−06	11.29/12.84%
5/2800	12.48/13.76%	9.8E−03/2.1E−08	12.56/13.86%
7/1600	8.40/10.08%	6.0E−02/2.5E−02	7.77/9.50%
7/2000	10.22/11.96%	3.2E−02/1.3E−04	9.82/11.65%
7/2400	11.10/12.68%	8.1E−03/1.5E−05	10.60/12.31%
7/2800	12.18/13.89%	1.6E−04/8.4E−08	11.96/13.77%

**Table 5:** Improvements of cBW + model selection, cBW alone vs Scheffer et al. in cases of correct initial transition matrix for synthetic data with posterior-Viterbi algorithm

State #/training sample	Average improvements of cBW + model selection in setting 1/2	Average *p* value of cBW + model selection in setting 1/2	Average improvements of cBW alone in setting 1/2
3/1600	7.46/8.01%	2.8E−02/1.7E−02	7.64/8.11%
3/2000	8.09/8.52%	3.0E−02/1.6E−02	8.25/8.60%
3/2400	8.32/8.67%	3.7E−02/5.9E−03	8.49/8.73%
3/2800	8.58/8.99%	4.9E−02/1.2E−03	8.79/9.09%
5/1600	9.52/10.62%	1.1E−01/5.1E−02	9.45/10.59%
5/2000	10.53/11.55%	6.0E−02/8.2E−03	10.47/11.63%
5/2400	11.51/12.58%	2.0E−02/3.5E−04	11.44/12.58%
5/2800	12.49/13.55%	1.7E−02/8.0E−06	12.55/13.61%
7/1600	8.75/10.19%	2.9E−02/2.6E−02	8.27/9.77%
7/2000	10.50/11.99%	2.1E−02/4.2E−03	9.82/11.31%
7/2400	11.15/12.47%	6.9E−02/7.8E−04	10.69/12.11%
7/2800	12.36/13.75%	5.2E−02/1.1E−05	12.05/13.57%

## Results

In this section, we set up experiments using both real biological data and synthetic data to test our method for decoding task and compared the results with those from using the method in [6]. It has been reported that [14, 15] posterior decoding in general performs better than Viterbi algorithm. So, in order to evaluate how our training method can impact on decoding, we carried out the decoding on the testing sequences with the trained model using both the standard Viterbi algorithm [10] and posterior-Viterbi algorithm described in [15], and the accuracy was computed by comparing the predicted label with the ground truth label at each position to determine the number of correct predictions:

$$\begin{aligned} Accuracy =\frac{ \# \,of\, correct\, predicted\, labels }{ \#\, of\, total\, labels} \end{aligned}$$

The results of these experiments show that our method outperforms Scheffer et al’s method in model training, as evidenced in the improved decoding accuracy, regardless which decoding algorithm is used. Specifically, on average, decoding accuracy improves by 33% with Viterbi algorithm, 36% with posterior-Viterbi algorithm in real data, and improves by 7.35–14.06% with Viterbi algorithm, 7.08–13.89% with posterior-Viterbi algorithm in synthetic data with significant *p* values. Note that, in two cases when the sequences are either almost fully labelled (95%) or very sparsely labelled (5%), the differences between various algorithms are insignificant. This phenomenon is no surprising though, as it is expected that the benefit from making good use of partial labels diminishes when labels are extremely sparse, which makes the various algorithms converge to Baum–Welch algorithm, or when sequences are almost fully labelled, which makes the various algorithms converge to the maximum likelihood. Therefore, our evaluations are divided into two settings for synthetic data. Setting 1 has partial label information from 5 to 95%. Setting 2 has partial label information from 10 to 90%.

### Synthetic data

The method described in [6] is mainly focused on handling text mining problems using synthetic data. To make the comparison fair, we have also performed experiments using synthetic data, which allowed us to observe our method’s different performance in different situations. In the experiments with synthetic data, the data is generated from ground truth HMMs, which are also generated randomly with predefined connections. For each experiment, the size for initial guess of transition and emission matrices are identical to the corresponding ground truth model. We fixed the number of symbols in hidden Markov model to be 20 to mimic the 20 amino acids in protein sequences. To test how model complexity may impact the training, we chose three different numbers of states: 3, 5, and 7. Moreover, different levels of training sample size were also considered as an experimental variable. Each experiment (with fixing state number and training examples) was evaluated for different levels of partial label and repeated for 50 times, and the corresponding paired *p* values were also calculated to assess the statistical significance of the performance difference between our method and the other method. Since our method can maintain the topology of initial guess of transition matrix, experiments were divided into two different groups. One was initialized the transition matrix with the same connectivity as the ground truth model, and the other was initialized with fully connected transition matrix.

**Fig. 2:**
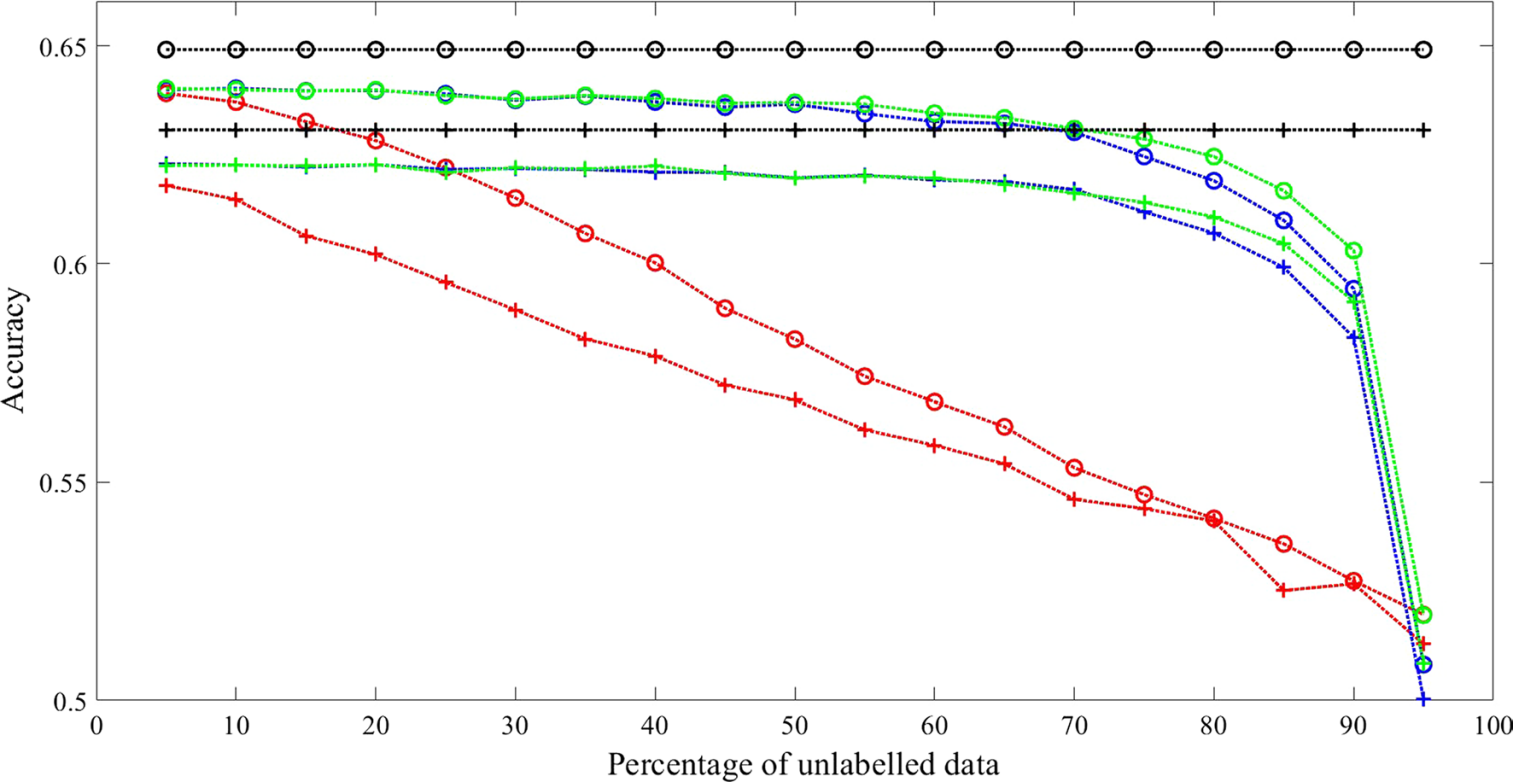
Comparison results 1 with synthetic data when initialized with fully connected transition matrix state number = 3 and training sample size = 1600. Training Methods: Ground truth model—black; cBW—green; cBW + model_selection—blue; Scheffer et al.—red. Decoding Methods: Viterbi—cross tick mark; posterior-Viterbi—circle tick mark. Generated by Matlab 2020a

**Fig. 3:**
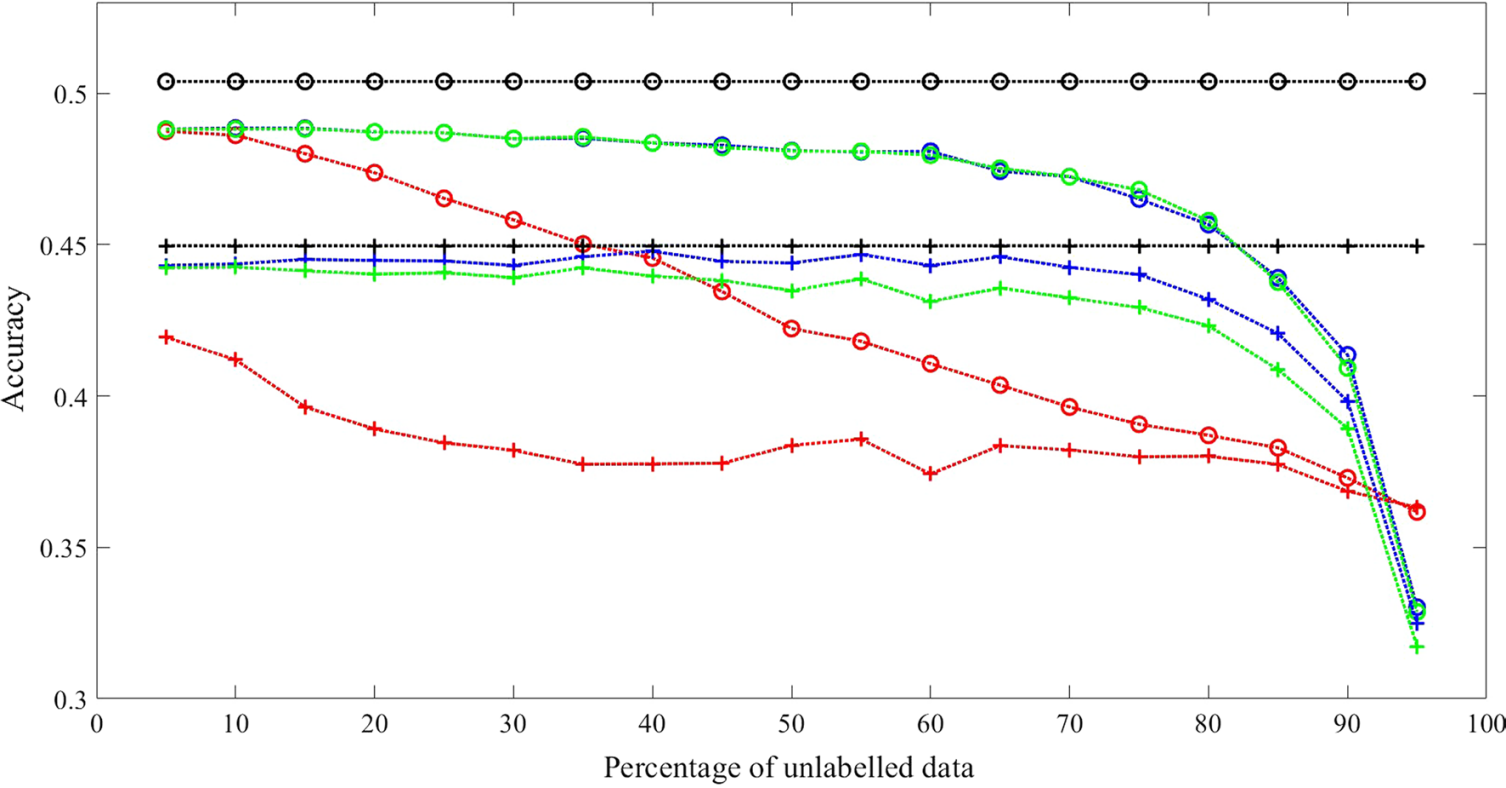
Comparison results 2 with synthetic data when initialized with fully connected transition matrix State number = 5 and training sample size = 1600. Training Methods: Ground truth model—black; cBW—green; cBW + model_selection—blue; Scheffer et al.—red. Decoding Methods: Viterbi—cross tick mark; posterior-Viterbi—circle tick mark. Generated by Matlab 2020a

**Fig. 4:**
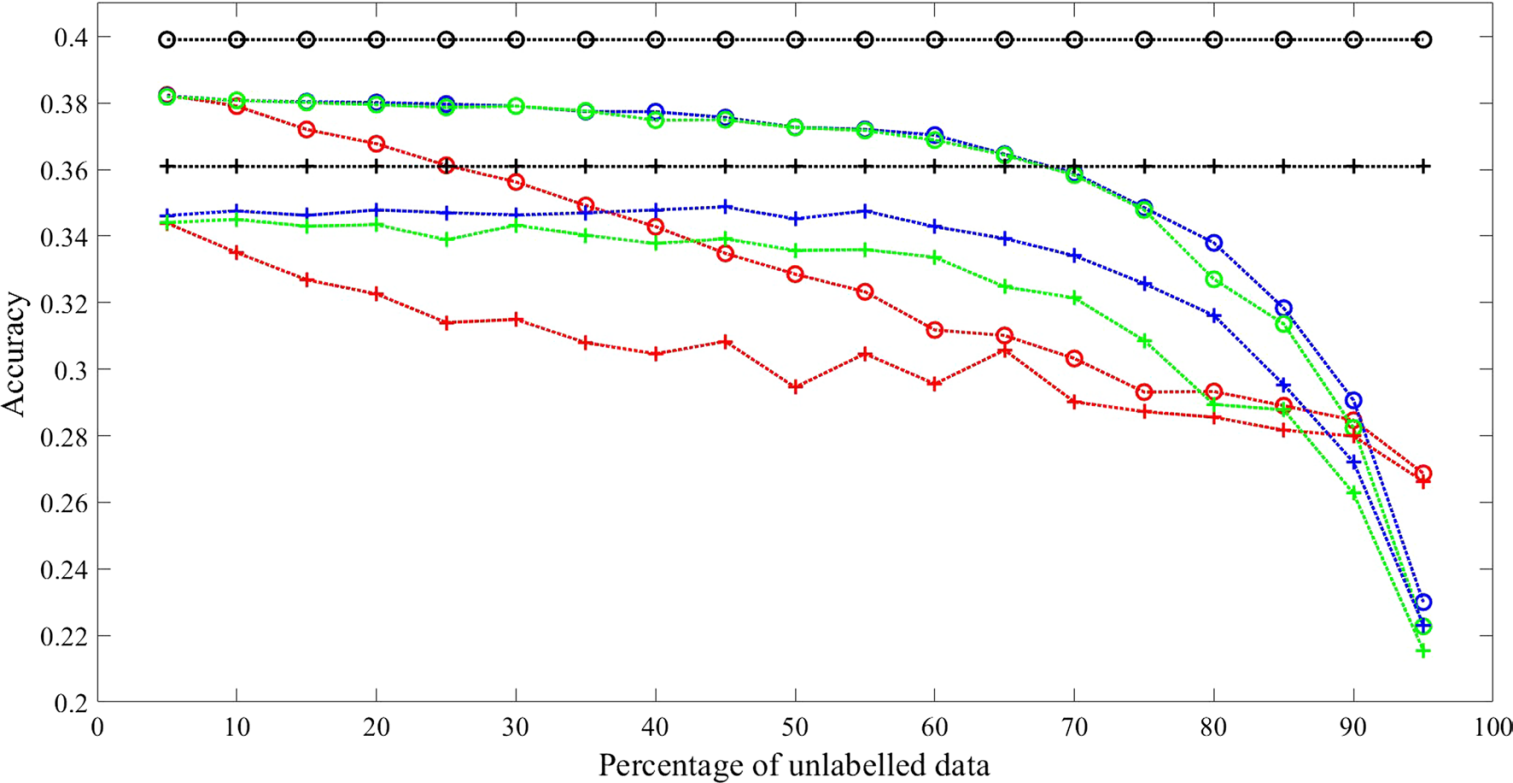
Comparison results 3 with synthetic data when initialized with fully connected transition matrix State number = 7 and training sample size is 1600. Training Methods: Ground truth model—black; cBW—green; cBW + model_selection—blue; Scheffer et al.—red. Decoding Methods: Viterbi—cross tick mark; posterior-Viterbi—circle tick mark. Generated by Matlab 2020a

Three sets of experimental results with fully connected transition matrix as initial guess are shown in Figs. 2, 3 and 4. Additional results are shown in Tables 2, 3, 4 and 5 for comparison.

Conducted using different numbers of states, training examples, and different decoding algorithms, the results show that our method outperforms the method by Scheffer et al. by 7.08–14.06% across different percentage of unlabelled data, with significant *p* value (< 0.05) for majority of the experiments. While both methods achieve a performance closer to that of the ground truth model as the level of partial labels increases, the improvement of our method over the method of Scheffer et al’s is more pronounced when partial labels are sparse, namely the level of unlabelled data is high, as shown in the X-axis of the Figures. For example, in Fig. 2, with Viterbi decoding, at the level of 70% unlabelled data, i.e., 30% partial labels, our method reaches an accuracy of 62%, which is 98% of the ground truth model accuracy, whereas Scheffer et al’s reaches accuracy of 54%, which is 85% of the ground truth model accuracy. Similar trends hold true for Figs. 3 and 4 when the model has 5 and 7 states respectively regardless of the decoding algorithm used.

### Real data

**Fig. 5:**
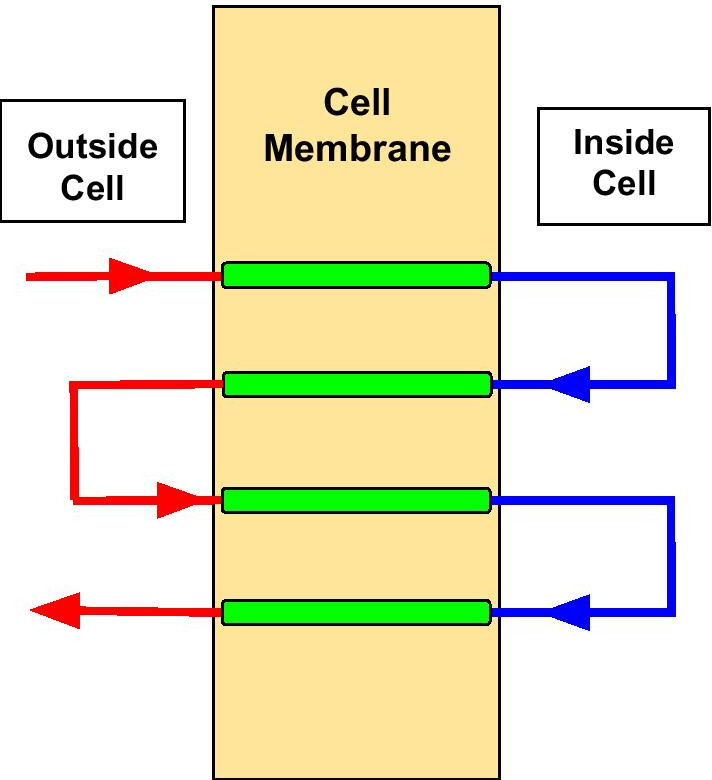
multi-pass transmembrane proteins the red lines represents protein sequence outside of cell membrane, the blue lines represents protein sequence inside of cell membrane, and green line represents transmembrane domain of the protein sequence. Generated by Google Drawings

**Fig. 6:**
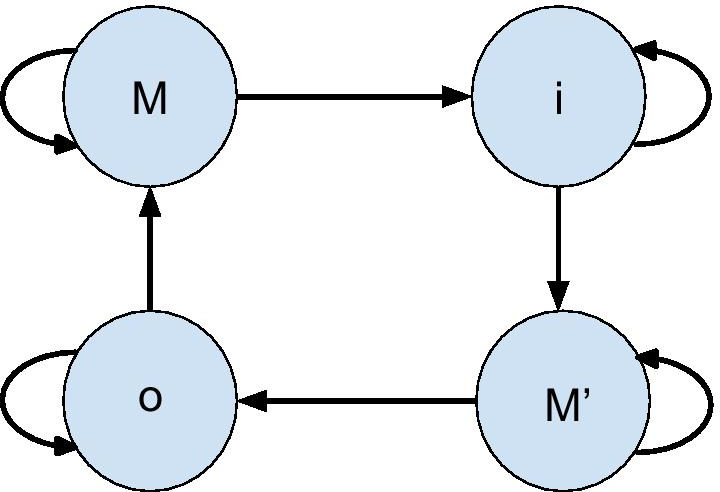
Topology of 4-state HMM for multi-pass transmembrane prediction States **i** and **o** represent inside and outside cell membrane respectively. Both **M** and **M**$^\prime$ stand for transmembrane domain, the redundant **M**$^\prime$ is used to avoid direct connection between state **i** and **o**, which is impossible. Generated by Google Drawings

For the real biological data, we adopted data from [16]. The data contains 83 multi-pass transmembrane proteins with complete label information. The topology of multi-pass transmembrane protein is shown in Fig. 5. The label for each sequence contain three different values: **i**, **o**, **M**. They stand for the region of protein sequence inside, outside cell membrane, and the transmembrane domain respectively. While much more sophisticated hidden Markov models have been used for modeling transmembrane protein topology [16–19], a simple HMM is used in this study to primarily evaluate the new training algorithm for partial labels. The architecture of the HMM is shown in Fig. 6, in which a redundant **M**$^\prime$ node is introduced as a simple mechanism to avoid a state path, such as **iiiimmmmiii** or **oooommmoooo**, that does not correspond to the real topology of transmembrane protein, in which a membrane domain has to be flanked by **i** on one side and **o** on the other side. Therefore, the transition matrix is 4 by 4, corresponding to the four states. Note that the amino acid emission frequencies for the transmembrane state are calculated by lumping together counts or expectation from both M and M’ states. We set up two different experiments with different initial conditions: (1) Transition matrix has correct zeros as ground truth model. (2) Transition matrix is fully connected. We set up experiments for condition (2) because the method in [6] cannot enforce initial zeros to remain zeros during the training, therefore, condition (2) gives more fair comparison of the two methods when no prior knowledge is available. The HMM is trained by these two different methods in a 10-fold cross validation scheme. Different levels of unlabelled data in training examples are actuated by selecting locations randomly to be unlabelled for each sequence. Since no ground truth model is available, maximum likelihood method with fully labelled training data is used to mimic the role of the ground truth model in experiments with synthetic.

**Fig. 7:**
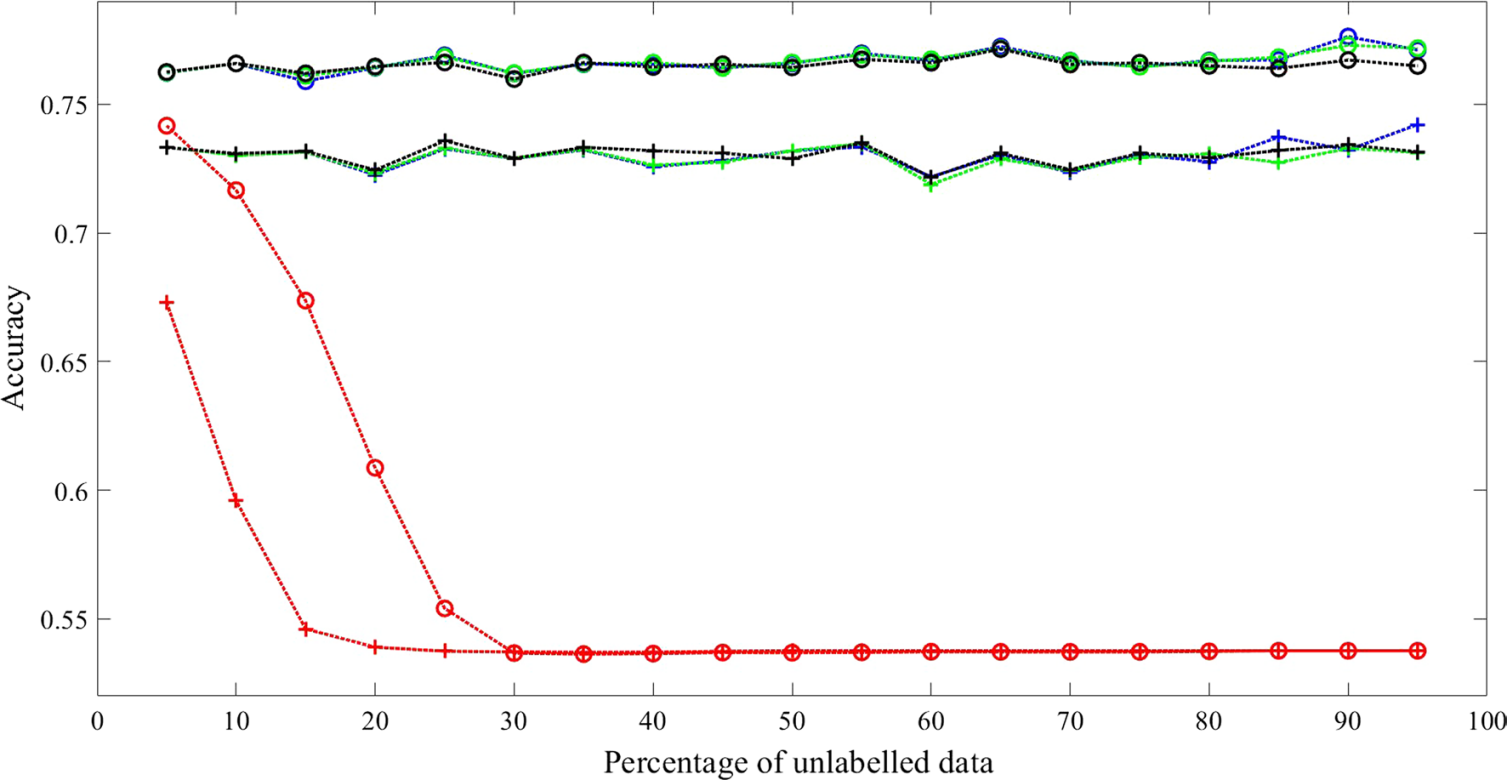
Comparison with real data when initialized with transition matrix of the correct connectivity in Fig. 5. Training Methods: ML—black; cBW—green; cBW + model_selection—blue; Scheffer et al.—red. Decoding Methods: Viterbi—cross tick mark; posterior-Viterbi—circle tick mark. Generated by Matlab 2020a

**Fig. 8:**
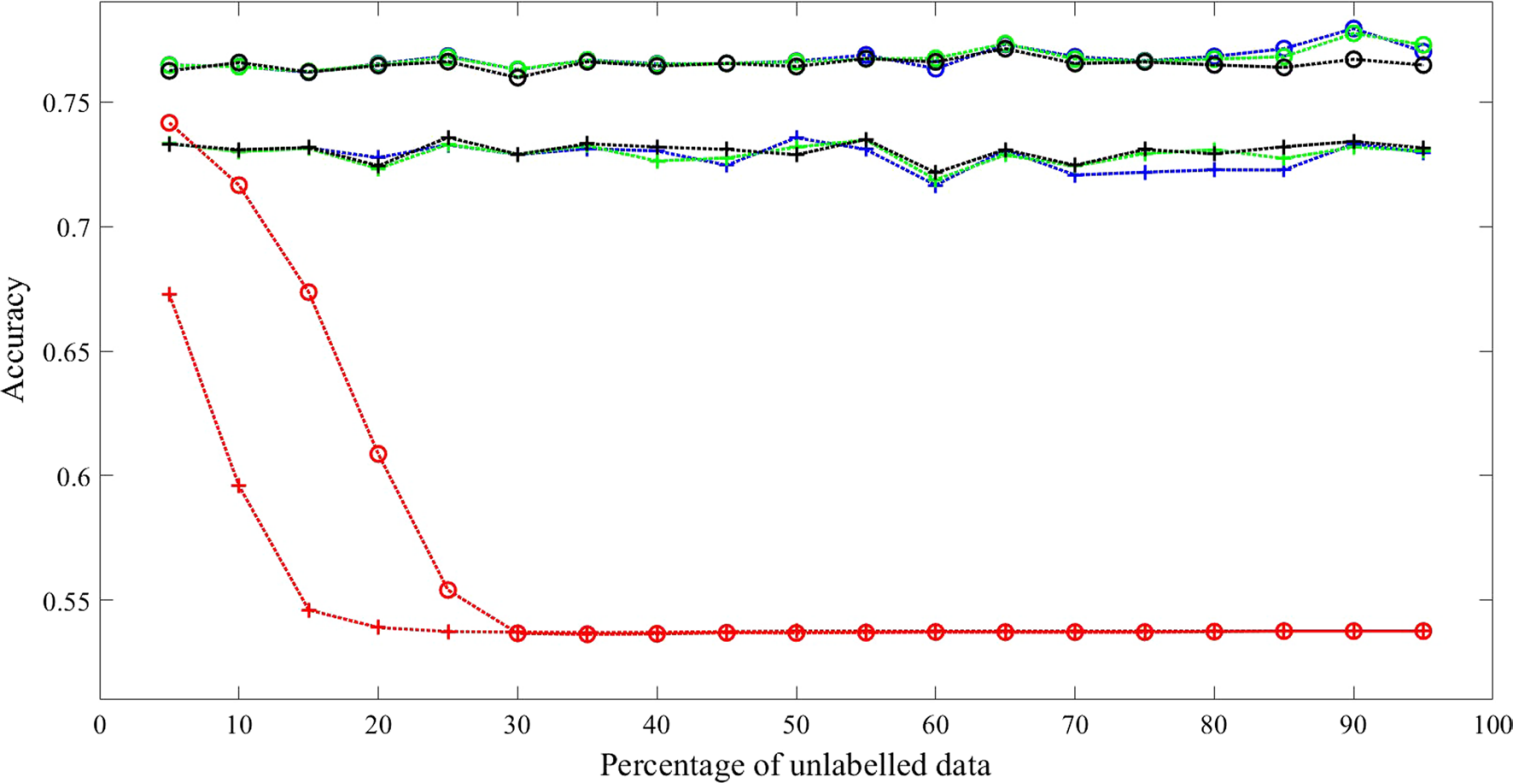
Comparison results with real data when initialized with fully connected transition matrix. Training Methods: ML—black; cBW—green; cBW + model_selection—blue; Scheffer et al.—red. Decoding Methods: Viterbi—cross tick mark; posterior-Viterbi—circle tick mark. Generated by Matlab 2020a

For condition (1), the result shown in Fig. 7 demonstrates that our method (constrained Baum–Welch with model selection) outperforms other method (Scheffer et al) by 33.59% with Viterbi Algorithm and 36.16% with posterior-Viterbi algorithm. For condition (2), the result shown in Fig. 8 attests that our method outperforms other method by 33.20% with Viterbi Algorithm and 36.32% with posterior-Viterbi algorithm. For both conditions, the performance of our method with or without model selection technique and maximum likelihood are very close.

## Discussion

From the results of experiments with synthetic data in Tables 2, 3, 4 and 5, they show: (1). constrained Baum–Welch algorithm with or without model selection technique achieve significant better performance than Scheffer et al. [6]; (2). constrained Baum–Welch benefit from having correct topology (comparisons between the 4th columns of Tables 2, 3); (3). constrained Baum–Welch algorithm performs better when model selection technique is used, especially when the task is hard (comparisons between 2nd and 4th column in Tables); (4). disregarding the training methods, posterior-Viterbi always outperforms standard Viterbi for decoding (Shown in Figs. 2, 3, 4, 7, 8).

From the results of experiments with real data, performance of constrained Baum–Welch with or without model selection are very close to maximum likelihood approach across different percentages of partial label. However, the performance of Scheffer et al’s drops dramatically after the percentage of unlabelled data is greater than 10%. The reason behind this is the method by Scheffer et al. cannot enforce the correct topology even the initial guess is correct. For this problem in particular, have a HMM with correct topology is key for higher accuracy.

Moreover, there are a few points worth mentioning for the benefits of those who may consider using this method for their applications. First, the ability of keeping correct topology makes cBW method compatible with more complex HMM, such as profile HMMs. However, as a trade-off, the training time can significantly increase. Second, model selection technique, although optional, is highly recommended to be used with posterior-Viterbi instead of standard Viterbi for best decoding performance. Lastly, our method is designed especially for the task of detecting de novo targeting signals, which assumes no fully labelled sequence is available in general. For the cases with relaxing constraints: some fully labelled sequences are available, our method is not the only choice, interested readers may also consider methods in [9].

## Conclusion

In this work, by modifying the standard Baum–Welch algorithm, we developed a novel training method, which, along with a model selection scheme, enables leveraging the partial labels in the data to improve the training of hidden Markov models. Compared with a similar method, our method achieved significant improvements in training hidden Markov models as evidenced by better performance in decoding both synthetic data and the real biological sequence data.

For future work, we will further investigate the impact of this training method on detecting de novo motifs and signals in biological data. In particular, we plan to deploy the method in active learning mode to the ongoing research in detecting plasmodesmata targeting signals and assess the performance with validations from wet-lab experiments.

## Data Availability

Datasets and source code are freely available on the web at https://www.cis.udel.edu/~lliao/partial-label-HMMs

## Abbreviations

HMM: Hidden Markov model
PDLP: Plasmodesmata-located proteins
cBW: constrained Baum–Welch algorithm
EM: Expectation–maximization
